# Road traffic injuries in Southeast Asia: making journeys safer

**DOI:** 10.1016/j.lansea.2024.100506

**Published:** 2024-11-08

**Authors:** The Lancet Regional Health – Southeast Asia

Around 1.19 million road traffic deaths are reported every year worldwide. On the World Day of Remembrance for Road Traffic Victims (occurring every third Sunday in November), we must rethink our efforts in reducing road traffic deaths because—in a fraction of a second—people’s lives change, with no option of a rewind button.

Road safety is the core of UN Sustainable Developmental Goal targets 3.6 and 11.2. In 2020, the UN's Decade of Action for Road Safety 2021–2030 called for reducing road traffic deaths and injuries by 50%. But we are far off target. According to the recent WHO South-East Asia Region (SEAR) status report on road safety, there were an estimated 330,222 road traffic deaths in 2021 in the region. Worryingly, there was only a 2% reduction from 2010 to 2021 and no difference between 2016 and 2021. Furthermore, in 2021 compared with 2010, WHO estimated that annual road traffic deaths had increased in Nepal by 25%, in Bangladesh by 23%, and India by 2%. The number of motorised two- or three-wheelers has also increased, especially in Indonesia, Sri Lanka, and Thailand, making prevention efforts more complex.

Considering multiple factors lead to road accidents, efforts at individual and environmental levels are necessary to reduce the risk of injury or death. Individual behaviours such as wearing a helmet and adhering to traffic rules are important. Police have a key role to play in reducing road traffic deaths by continuously monitoring the vehicle speeds and conducting unbiased routine inspections. It would be helpful to reduce the burden of legal paperwork, so that police could prioritise transportation of accident victims to the nearest health centre. Driving under the influence of alcohol and drugs significantly increases the risk of road traffic deaths. Corruption and lengthy legal systems allow influential people to get away with their crimes fostering an environment of recurrent offences and noncompliance with rules and regulations. Countries in SEAR could invest in mandatory body cameras for police similar to close-circuit television cameras used for monitoring traffic. However, understaffed departments and inadequate training, becomes obstacles to regular inspections by the police. Recently, a shocking bus accident in Thailand led to the deaths of more than 20 school children due to negligent monitoring by police and the motor vehicle department. The families of the deceased have called for stricter monitoring of vehicle safety.

Well maintained roads with traffic signs can avert deaths. A 2017 study from Bangladesh showed that additional speed bumps, road signs, and markings led to a significant decrease (66%) in road accidents and injuries. Prompt warnings to citizens about floods and cyclones would help save lives too. However, corruption, poor regulation, overloaded vehicles, and climate change are a deadly combination leading to damaged roads. For example, roads are deliberately repaired during rainy days, resulting in poorly engineered roads eroding faster. Also, laws related to mandatory safety measures such as using good quality helmets, child restraint measures, seat belts, etc are sometimes heavily resisted by the public (owing to cost, lack of awareness, and high risk behaviour), posing a major challenge.

Public awareness and training can play a major role and go a long way in saving people’s lives. Education departments can include training in first aid and cardiopulmonary resuscitation in school curriculums. Governments can promote public awareness videos via television or social media. For example, pedestrians, especially children and elders, could learn to avoid the blind spots of buses and trucks. In SEAR, only India has a Good Samaritan Law, which protects people helping accident victims from legal harassment. The University Grants Commission of India is actively promoting awareness about the Good Samaritan Law among university students.

Among the SEAR countries, Thailand has commendably addressed the issue of under-reporting and has reduced the gap between its officially reported road traffic deaths and the WHO estimates. Studies from Bangladesh and Nepal have shown that local record keepers or shopkeepers can help in recording accidents. These data could be compared with police records to improve reporting. For SEAR, the target of halving road traffic deaths and injuries by 2030 may be too ambitious. But on a positive note, from 2010 to 2021, there were decreases of 46% and 42% in estimated road traffic deaths in Maldives and Thailand, respectively, which may be attributed to promotion of road safety practices. With continued efforts along with better responsibility and accountability, we can make our journeys safer.

